# Cytochrome 450 1B1 (CYP1B1) polymorphisms associated with response to docetaxel in Castration-Resistant Prostate Cancer (CRPC) patients

**DOI:** 10.1186/1471-2407-10-511

**Published:** 2010-09-27

**Authors:** Ilaria Pastina, Elisa Giovannetti, Aldo Chioni, Tristan M Sissung, Francesco Crea, Cinzia Orlandini, Douglas K Price, Claudia Cianci, William D Figg, Sergio Ricci, Romano Danesi

**Affiliations:** 1 Department of Medical Oncology, Pisa University Hospital, Pisa, Italy; 2 Grosseto Civic Hospital, Grosseto, Italy; 3 Division of Pharmacology and Chemotherapy, Department of Internal Medicine, University of Pisa; Via Roma 55, 56100 Pisa, Italy; 4 Department of Medical Oncology, VU University Medical Center, De Boelelaan 1117, 1081HV, Amsterdam, The Netherlands; 5 Medical Oncology Branch, National Cancer Institute, 9000 Rockville Pike Building 10, Room 5A01, Bethesda, MD 20892, US

## Abstract

**Background:**

The selection of patients according to key genetic characteristics may help to tailor chemotherapy and optimize the treatment in Castration-Resistant Prostate Cancer (CRPC) patients. Functional polymorphisms within the *cytochrome P450 1B1 *(*CYP1B1*) gene have been associated with alterations in enzymatic expression and activity and may change sensitivity to the widely used docetaxel regimen.

**Methods:**

*CYP1B1 *genotyping was performed on blood samples of 60 CRPC patients treated with docetaxel, using TaqMan probes-based assays. Association between *CYP1B1*-142C>G (leading to the 48ArgGly transition), 4326C>G (432LeuVal), and 4390A>G (453AsnSer) polymorphisms and treatment response, progression-free-survival (PFS) and overall-survival (OS) was estimated using Pearson χ^2 ^test, Kaplan-Meier curves and Log-rank test.

**Results:**

Patients carrying the *CYP1B1*-432ValVal genotype experienced a significantly lower response-rate (P = 0.014), shorter progression-free-survival (P = 0.032) and overall-survival (P < 0.001). Multivariate analyses and correction for multiple comparisons confirmed its prognostic significance for OS. No significant associations were found among other polymorphisms and both response and clinical outcome.

**Conclusions:**

*CYP1B1*-4326C>G (432LeuVal) polymorphism emerged as possible predictive marker of response and clinical outcome to docetaxel in CRPC patients and may represent a potential new tool for treatment optimization. Larger prospective trials are warranted to validate these findings, which might be applied to the future practice of CRPC treatment.

## Background

Prostate cancer is the most common malignancy in men and the second leading cause of cancer death among males in the Western World [1]. Approximately 70 to 80% of patients with advanced prostate cancer respond to medical or surgical castration [2]. When tumours become refractory to androgen withdrawal therapy, most systemic treatments offer modest benefit in terms of overall-survival (OS) [3].

Two multicenter phase III randomized clinical trials, TAX 327 and SWOG 9916, showed a survival advantage in Castration-Resistant Prostate Cancer (CRPC) patients treated with taxane-based chemotherapy [4,5]. In both trials the rates of prostate-specific-antigen (PSA) response and OS were significantly higher in the docetaxel groups compared to mitoxantrone and prednisone. In particular, PSA responses were 45% and 50%, while OS was 18.9 and 17.5 months in docetaxel-treated patients, in the TAX 327 and in the SWOG 9916 trial, respectively. On the basis of these findings, taxane-based chemotherapy is now considered the standard first-line therapy for CRPC patients.

Despite the relative success in the treatment of these patients, high variability in the clinical response to docetaxel has been observed [6]. This variability can be partially attributed to a poor understanding of inter-individual differences in the pharmacokinetics and pharmacodynamics of docetaxel. Due to the clinical relevance of docetaxel, genetic markers with predictive power to assess inter-patient differences in clinical response are urgently needed.

There is evidence from preclinical and clinical studies that the cellular response to docetaxel is linked to expression and/or activity of cytochrome-P450 1B1 (CYP1B1). Although docetaxel is not directly metabolized by CYP1B1 [7,8], CYP1B1 overexpression resulted in a significant decrease in docetaxel sensitivity in transfected hamster ovary cells [9]. CYP1B1 was also induced by docetaxel treatment as a mechanism of resistance in several breast cancer cell lines; the same effect was not observed in breast tumours following treatment with other drugs, nor was it observed for docetaxel applied to cell lines derived from tumours without significant hormone-related etiology [10]. Recent evidence suggests that this is probably due to the action of 4-hydroxyestradiol, the major CYP1B1 metabolite. The 4-OHE2-derived quinone has been shown to impair docetaxel antitumour activity through both an interference with the microtubule stabilization induced by docetaxel, and by direct structural alteration of docetaxel [11].

CYP1B1 activity is regulated by several functional non-synonymous single-nucleotide-polymorphisms (SNPs). The 4326C>G SNP (rs1056836), leading to the 432LeuVal (432LV) amino-acid transition, is associated with increased catalytic activity of CYP1B1 [12,13], while the 142C>G SNP (rs10012), leading to 48ArgGly (48RG) transition, resulted in increased *CYP1B1 *gene expression [12,14], without alterations in catalytic properties unless in combination with other functional alleles. Finally, the 4390A>G SNP (rs 1800440), leading to 453AsnSer (453NS) transition, has been associated with decrease in protein expression due to an increase in CYP1B1 degradation [15].

Interestingly, the homozygous variant of the 4326C>G SNP (CYP1B1*3-allele) has been associated with enhanced synthesis of 4-OHE2, increasing the intracellular ratio with the minor CYP1B1 metabolite 2-OHE2 [8], and CRPC patients carrying two copies of CYP1B1*3 had a significantly shorter OS after docetaxel-based therapies [11].

However, no data on response were reported, and further studies are needed to clarify the role of *CYP1B1 *genotype and docetaxel activity in CRPC patients. For this purpose we retrospectively evaluated the correlation between the 142C>G, 4326C>G and 4390G>A SNPs and clinical outcome in 60 docetaxel-treated CRPC patients.

## Methods

### Patient selection criteria

The present study was performed in CRPC patients treated with docetaxel between January 2005 and March 2007 in the Division of Oncology of the S.Chiara-University-Hospital (Pisa, Italy). Patients of age > 18, performance status 0-3 (ECOG scale) with histologically confirmed diagnosis of prostate cancer, were eligible for the study. Other eligibility criteria were hormone refractory disease, ANC-count ≥ 1.5 × 10^3^/ml, platelet-count > 10^5^/ml, Hb > 10g/dl and life-expectancy > 3 months, while exclusion criteria included hypersensitivity to docetaxel and inadequate cardiac/epatic/renal function. The Ethics Committee of S.Chiara-University-Hospital approved the protocol. Patients were required to sign an informed consent before their enrolment.

### Treatment

All patients received docetaxel given intravenously over 1-hour at a dose of 75 mg/m^2 ^on day-1 every 21 days or docetaxel 30 mg/m^2 ^weekly for five of every six weeks plus prednisone 10 mg os daily.

### Evaluation criteria

The primary endpoint of this analysis was the correlation of the candidate polymorphisms with antitumour response as determined by the effect of treatment on PSA concentration, whereas secondary endpoints included correlation with progression-free-survival (PFS) and overall survival (OS). Serum PSA and physical examination were evaluated every three weeks while CT imaging and bone scan were performed at baseline and every three cycles. A response was defined as a reduction from baselines of at least 50% that was maintained for at least three weeks, whereas PSA progression was defined as an increase from the nadir of either at least 25% for men with no PSA response or at least 50% for all others. Stable disease was defined as an increase from the nadir less than 25% for men with no PSA response or less than 50% for all others. PFS was defined from the date of randomization to the date of PSA progression as an increase ≥ 25% in PSA level from baseline or ≥ 50% from the lowest value achieved; the PSA increase should be at least 5 ng/ml confirmed by three measurements at 3 week intervals. Other criteria of progressive disease were the appearance of a new lesion or a ≥ 25% increase using standard bidimensional measurements, in accordance with WHO guidelines, of previously measured disease.

### DNA isolation

Genomic DNA was extracted from blood samples (5 ml) drawn before drug administration, using the QIAmp-DNA-mini-Kit (Qiagen, Hilden, Germany). DNA yields and integrity were checked by optical density at 260 nm, while testing for contamination was performed by measuring absorbance at 280 nm and calculating the 260/280 ratio.

### Polymorphisms analysis

*CYP1B1 *polymorphisms at 48ArgGly, 432LeuVal and 453AsnSer were studied with Taqman^®^-probes-based assays using the ABIPRISM-7900HT instrument equipped with the Sequence-Detection-System *version *2.0 software (Applied Biosystems, Foster City, CA). Forward (F) and reverse (R) primers and probes (P) were obtained from Assay-on-Design-SNPs products, using the File-Builder software *version *2.0 (Applied Biosystems), i.e. for 142C>G: (F) 5'-GCT GCT GAG GCA ACG GA-3'; (R) 5'-CAG TGG CCA CGC AAA CG-3'; (VIC-P) 5'-CAG CTC CGG TCC GC-3' (FAM-P); 5'-AGC TCG GGT CCG C-3' for 4326C>G: (F) 5'-CAG CTC GAT TCT TGG ACA AGG A-3'; (R) 5'-TGC CCA CTG AAA AAA TCA TCA CTC T-3'; (VIC-P) 5'-CCT CAT CAA CAA GGA C-3'; (FAM-P) 5'-CTC ATC AGC AAG GAC-3'; and for 4390A>G: (F) 5'- TTT GTC AAC CAG TGG TCT GTG AAT-3'; (R) 5'-GGA TCA AAG TTC TCC GGG TTA GG-3'; (VIC-P) 5'-CAT GAC CCA CTG AAG TG-3'; (FAM-P) 5'-ATG ACC CAG TGA AGT G-3'.

PCR reactions were performed using 20 ng of DNA diluted in 11.875 μl DNAse/RNAse-free water, 12.5 μl of TaqMan Universal-PCR-Master-Mix, with AmpliTaq-Gold^®^, and 0.625 μl of the assay mix (F and R primers and the specific probes), in a total volume of 25 μl. After thermal cycling (40 cycles of denaturation at 95°C for 15 seconds, followed by annealing and extension at 60° for 1-minute), the instrument determined the allelic content of each sample by reading the generated fluorescence, as described [16].

### Statistics

This study was aimed at evaluating the correlation of selected *CYP1B1 *SNPs with response to docetaxel, while the secondary endpoints included the correlation with PFS, and OS.

Demographic and clinical information on response were compared across genotype, using Pearson-χ^2 ^test. Analyses included information on genotypes, ECOG-performance status (PS, 0 vs. > 0), age (≤ median vs. > median), pre-treatment PSA (≤ median vs. > median), visceral metastatic disease (yes vs. no), anaemia (eg, Hb < 13.0 g/dl, yes vs. no), and schedule (3 week vs. weekly administration). PFS was calculated from the start of chemotherapy to the date of clinical and/or biochemical and/or radiological evidence of progression or death, whichever occurred first. OS was calculated from the day of treatment start to the endpoint (death or last follow-up). The Kaplan-Meier method was used to plot PFS and OS and the Log-rank test to compare curves. Known baseline prognostic variables were also included in multivariate analyses, using Cox's proportional hazards model to identify factors of independent significance. A step-down procedure was used based on the likelihood ratio test, and hazard ratios were calculated to estimate the magnitude and the direction of the effect.

Data were analyzed using *SPSS/PC+17 *software (SPSS Inc., Chicago, IL). Statistical significance was set at *P *< 0.05. However, in the univariate analyses for clinical outcome according to the 3 studied polymorphisms, a Bonferroni correction required a P < 0.05/3 = 0.016 for statistical significance.

## Results

### Patient characteristics, treatment and clinical response

A total of 60 consecutive patients were enrolled in this study. Clinical characteristics are listed in Table 1. All patients had CRPC with metastatic evaluable disease. Median pre-treatment PSA value was 53.5 ng/ml (range 5.55-6024.00). All patients received docetaxel as first-line treatment, administered every 3 weeks in 80.0% of the patients, while 20.0% of them received docetaxel weekly. Median cycles administered in all patients were 6 (range, 1-10). No toxic death occurred. Since the incidence of grade-3 neutropenia was low and grade-4 toxicity was rare (1 case), no dose reduction was required. All patients were evaluable for PSA response. Six patients had CR, 23 PR, 13 SD and 18 PD. Median PFS was 7.1 months (95% CI, 5.6-8.5), while median OS was 20.4 months (95% CI, 16.6-24.2).

**Table 1:** Characteristics of CRPC patients

	N (%)
**All Patients**	60
**Age, median (range)**	71 years (58-84)
**< 71**	31 (51.7)
**> 71**	29 (48.3)
**ECOG PS**	
**0**	51 (85.0)
**1-3**	9 (15.0)
**Schedule**	
**Weekly**	12 (20.0)
**3 weeks**	48 (80.0)
**Visceral metastasis**	
**yes**	17 (28.3)
**no**	43 (71.7)
**Anaemia**	
**yes**	27 (45.0)
**no**	33 (55.0)

### CYP1B1 polymorphisms

All the samples were evaluable for *CYP1B1 *SNPs. For the 4326C>G *CYP1B1*, the frequencies of CC, GC and GG genotypes were 20%, 42% and 38%, respectively. The wild-type *CYP1B1 *4390AA had a frequency of 62%, while the heterozygous 4390AG and homozygous 4390GG variants had a frequency of 30% and 8%, respectively. Regarding *CYP1B1 *142CG, the CC variant was found in 13% of cases, while the heterozygous CG and the GG variants were observed in 35% and 52% of cases, respectively.

All polymorphisms followed Hardy-Weinberg's equilibrium as calculated with the *SNP- analyzer-software *(P = 0.42, 0.31 and 0.46 for 4326C>G, 4390G>A and 142C>G *CYP1B1 *SNP, respectively). Genotype frequencies of 4326C>G allele were comparable (P > 0.05, χ^2^-test) with those reported in a study in a Caucasian population of prostate cancer patients [17], while the frequencies of 142C>G and 4390A>G were similar to those observed in control Caucasian populations [18,8]. Patients and genotype characteristics were well balanced between the 2 schedules of treatment. No significant correlations were detected between genotype and the other patient's characteristics.

### Correlation between clinical characteristics and outcome

The overall response rate (CR+PR = RR) of the patients was 48.3%. Age, ECOG-PS, docetaxel-schedule, pre-treatment PSA and visceral metastasis were not associated with response (P = 0.443, 0.914, 0.846, 0.605 and 0.325 respectively), whereas anaemia was significantly associated with response (22 out of the 29 responding patients (eg, 75.9%) have Hb ≥ 13.0 g/dl, P = 0.004).

None of the clinical characteristics was associated with PFS, while the occurrence of visceral metastasis and anaemia were associated with significantly shorter OS. In particular, median OS was 15.8 (95%CI, 12.4-19.1) months in patients with Hb < 13.0 g/dl vs. 28.3 (95%CI, 16.9-39.7) months in patients with Hb ≥ 13.0 g/dl (P = 0.006). Similarly, patients with visceral metastasis had a median OS of 13.9 (95%CI, 10.8-16.9) months, while patients without visceral metastasis had a median OS of 23.2 (95%CI, 16.4-30.0) months (P = 0.001).

Finally, responding patients had a significantly longer OS (28.9 (95%CI, 27.5-30.3) vs. 15.4 (95%CI, 12.0-18.8) months, P < 0.001) and PFS (8.4 (95%CI, 7.8-8.9) vs. 4.1 (95%CI, 2.1-6.1) months, P = 0.009) than patients with SD or PD.

### Correlation between genetic polymorphisms and outcome

When grouping patients between those with response (CR and PR) and without response (SD and PD), and between those with genotype leading to increased CYP1B1 activity or expression (4326GG or 142GG) and with genotype leading to reduced CYP1B1 expression (4390AG or 4390GG), no significant correlations with response were observed for the *CYP1B1 *polymorphisms at position 4390 and 142 (Table 2). On the contrary, a significant correlation was demonstrated between response to docetaxel and *CYP1B*1 4326C>G polymorphism: 62.2% of the patients carrying the *CYP1B1 *4326CC and 4326CG genotype experienced CR or PR, whereas only 26.1% of the *CYP1B1 *4326GG patients responded to therapy (P = 0.014).

**Table 2:** Response according to *CYP1B1 *polymorphisms

	CR+PR (N = 29)	SD+PD (N = 31)	
	
Genotype	N (%)	N (%)	*P*
**CYP1B1 4326CG**			
**CC + GC**	23 (62.2)	14 (37.8)	0.014
**GG**	6 (26.1)	17 (73.9)	
**CYP1B1 4390GA**			
**AA**	21 (56.8)	16 (43.2)	0.164
**AG+ GG**	8 (34.8)	15 (65.2)	
**CYP1B1 412CG**			
**CC + CG**	14 (48.3)	15 (51.7)	0.993
**GG**	15 (48.4)	16 (51.6)	

Abbreviations: CR complete response; PD, progressive disease; PR, partial response; SD, stable disease. *P *was calculated with *Pearson-χ2 two-sided *test with continuity correction

Patients harbouring both the *CYP1B1 *4326GG and 412GG genotype (N = 9) also experienced a significant lower rate of response (P = 0.039), with respect to patients (N = 51) harbouring the other genotypes. Indeed, 8 out of 9 (88.9%) patients with the *CYP1B1 *4326GG and G412GG genotype experienced PD or SD, while 28 out of 51 (54.9%) patients harbouring the other genotypes achieved CR or PR. Furthermore, the patients carrying both the *CYP1B1 *4326GG and the 4390AG or 4390GG genotype (N = 13) experienced a significant lower prevalence of response with respect to patients (N = 47) harbouring the other genotypes (P = 0.003). Indeed, 12 out of 13 patients with the *CYP1B1 *4326GG and the 4390AG or 4390GG genotype experienced PD or SD, whereas 28 out of the 47 patients carrying the other genotyes achieved CR or PR. In contrast, no differences were observed grouping patients with the *CYP1B1 *4390AG or 4390GG and the 412GG genotype

**Figure 1:**
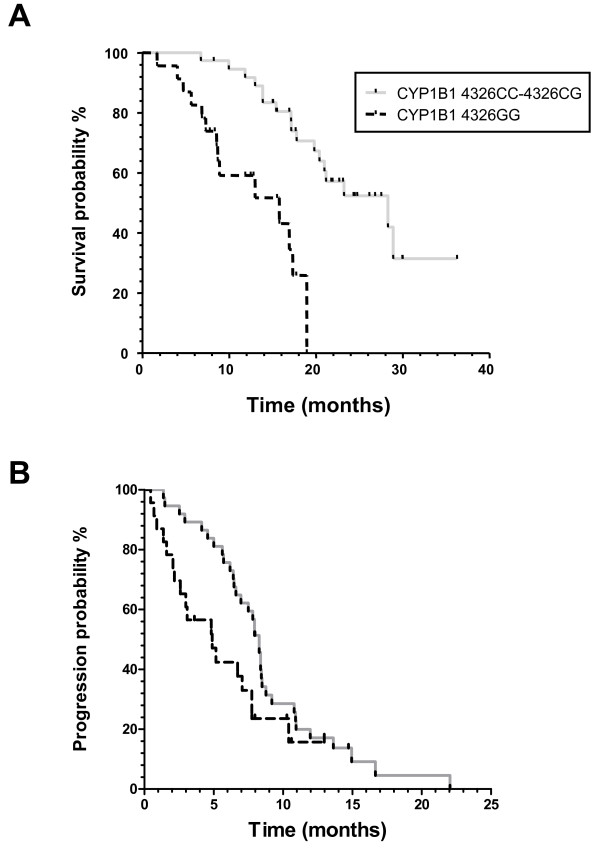
**Kaplan-Meier curves of OS (A) and PFS (B) according to *CYP1B1 *4326C>G polymorphism in the CRPC patients treated with docetaxel enrolled in the present study (N = 60)**.

However, the *CYP1B1 *4326GG genotype was associated with shorter PFS and OS (Fig. 1A-B). In particular, the median OS of patients harbouring the G/G variant was 15.8 months, while patients carrying the C/G or the C/C genotype had a median OS of 28.3 months, respectively (Fig. 1A). In order to keep at minimum the probability to find a statistically significant difference purely by chance, the usual nominal level (P = 0.05) has been lowered to 0.016 by Bonferroni adjustment for multiple comparisons considering the 3 studied SNPs. After this adjustment, no statistically significant differences were detected for PFS (P = 0.032), while the *CYP1B1 *4326GG genotype was still significantly correlated to OS (P < 0.001). The median age of the 23 patients harbouring the *CYP1B1 *4326GG was 69 years (range = 58-80). Nine (39%) of these patients had visceral metastasis, while 19 (83%) had anaemia. No significant correlations were observed between OS and PFS in patients grouped according to the other studied polymorphisms (Table 3). However, both the grouped *CYP1B1 *4326GG and 412GG patients and the grouped *CYP1B1 *4326GG and 4390GA or 4390GG patients had significantly shorter OS (P = 0.001 and 0.022, respectively), while no correlations were observed with PFS.

**Table 3:** PFS and OS according to *CYP1B1 *polymorphisms

	Median PFS			Median OS		
	
Genotype	Mos	95% CI	P	Mos	95% CI	P
**CYP1B1 4326CG**						
**CC + GC**	8.3	7.7-8.8	0.032	28.3	17.3-39.2	< 0.001
**GG**	4.8	2.0-7.6		15.8	5.3-26.3	
**CYP1B1 4390AG**						
**AA**	7.5	6.1-8.9	0.295	19.8	15.6-24.0	0.945
**AG+ GG**	7.0	2.6-11.3		10.4	11.3-29.5	
**CYP1B1 412CG**						
**CC + CG**	7.1	5.3-8.8	0.656	18.9	15.8-22.0	0.584
**GG**	7.5	5.9-9.1		21.2	13.2-29.1	

Abbreviations: Mos, months; NR, not reached OS, overall survival; PFS, progression-free-survival. P values were calculated with *log-rank *test.

Note. The event-rate was 51.7%, and 6 patients were alive without progression at the last follow-up (July 2008).

To evaluate the risk of death we carried out a Cox regression analysis entering known prognostic baseline factors (i.e. pre-treatment PSA, anaemia, visceral metastasis) and the polymorphisms significantly associated with OS in the univariate model (i.e. *CYP1B1 *4326C>G polymorphism). In this model, limited by the small sample size, we found that response and visceral metastasis were always associated with OS, while pre-treatment PSA and anaemia were not independent predictors of OS (Table 4). However, the *CYP1B1 *4326GG genotype resulted a significant predictor of OS (P = 0.003). In a second multivariate we also evaluated the grouped *CYP1B1 *4326GG and 412GG as well as the grouped *CYP1B1 *4326GG and 4390AG or 4390GG genotypes, and both these grouped genotypes showed a significant association with OS (P = 0.029 and P = 0.010, respectively).

**Table 4:** Multivariate analysis of clinical and biological parameters for OS

Covariates		HR (95% CI)	*df*	*P*
**Visceral metastasis**	**yes**	3.8 (1.4-10.0)	*1*	*0.008*
	**no**	1 (Ref.)		
**Anaemia**	**yes**	1.0 (0.2-2.5)	*1*	*0.935*
	**no**	1 (Ref.)		
**Pre-treatment PSA**	**≤ median**	1 (Ref.)	*1*	*0.242*
	**> median**	0.64 (0.3-1.4)		
***CYP1B1 *G4326C**	**4326CG+4326CC**	1 (Ref.)	*1*	*0.003*
	**4326GG**	1.7-12.8		

Abbreviations: Ref., Reference.

## Discussion

This study investigated selected *CYP1B1 *polymorphisms as potential biomarkers of activity/resistance to docetaxel in CRPC, and provides evidence that the *CYP1B1 *4326C>G polymorphism may be associated with significantly lower response rate and shorter PFS/OS after docetaxel treatment.

Therapeutic options against prostate cancer have been expanded considerably in the last decade by the introduction of docetaxel for the treatment of CRPC patients [4,5]. Docetaxel is a second-generation drug of taxane family, with marked activity against *in vitro *and *in vivo *models of prostate cancer, where it has been shown to induce apoptosis, inhibit angiogenesis and modulate expression of several signaling pathways [19]. The preclinical promise translated in clinical practice and docetaxel is effective as monotherapy and combination therapy across a variety of tumour types, including prostate cancer. The activity of docetaxel against CRPC has been confirmed in the present work by the percentage of PSA response and median OS, which were 48.3% and 20.4 months, respectively.

The reasons for therapeutic success or failure are, however, elusive and few studies have evaluated possible genetic markers to select patients most likely to respond to docetaxel.

A major mechanism of resistance to taxanes may be represented by the mutation/overexpression of tubulin. In particular, *β-tubulin-class-I *mutations were identified in 33% of 49 tissues from non-small-cell lung cancer (NSCLC) patients, none of whom had an objective response to paclitaxel [20], but we still lack the validation of mutational analysis or gene expression quantification of tubulin-isotypes as predictive tests to tailor treatment with taxanes.

Other studies have evaluated polymorphisms of the multidrug resistance (MDR1) gene, whose product P-glycoprotein functions as an ATP-dependent exporter of several drugs, including paclitaxel and docetaxel, from cells. Subjects homozygous for C in position 3435 (3435C>T) had higher *MDR1 *mRNA expression in leukocytes than subjects with the TT genotype [21]. This SNP together with the 2677G>A polymorphism, has been associated with better response in NSCLC patients treated with docetaxel-cisplatin chemotherapy, but no significant correlations were found with response in ovarian cancer patients treated with paclitaxel [22,23].

In CRPC patients receiving docetaxel alone, individuals carrying a diplotype consisting of the 1236C-2677G-3435C *MDR1 *linked alleles had significantly improved overall survival, but no data were available on response [24].

Assessing germline genetic polymorphisms as either predictive or prognostic markers is very appealing, especially in the CRPC patients. In these patients primary tumours, as well as bone metastasis, are not resected so that the avaibility of tumour material can be problematic. SNPs are inherited genetic variants harboured by all the cells and their analysis can be easily performed in blood and is easier to adopt in the routine clinical setting than tumour expression arrays, which need biopsies of patient's tumours, laser-microdissection and sophisticated infrastructure.

Therefore, in the present study we evaluated functional polymorphisms of *CYP1B1*, which play an important role in cancer risk and progression as well as in the metabolism of cancers modulated by sex hormones. In particular, estrogen exposure has been implicated in the disease aetiology of prostate cancer, and CYP1B1 is up-regulated in prostate cancer [25]. Previous studies showed that *CYP1B1 *polymorphisms may be involved in cancer risk, alone or in combination with other factors [8]. In particular, the 4326GG allele was associated with increased cancer incidence in a Caucasian population [17], where the prevalence of this genotype in CRPC patients (34%) was comparable to that observed in the present study (36%).

However, this study focused on the analysis of *CYP1B1 *polymorphisms in order to establish their possible relationships with clinical outcome in CRPC patients treated with docetaxel.

The primary end-point of this study was the correlation with response and significant response differences were observed according to *CYP1B1 *4326C>G polymorphism. Patients carrying the *CYP1B1 *4326CC or the 4326CG genotype experienced positive clinical response in a significantly higher rate than *CYP1B1 *4326GG patients.

Since previous studies reported a linkage-disequilibrium between the *CYP1B1 *4326GG and the 412GG polymorphisms [26], we also performed a statistical analysis in patients harbouring the *CYP1B1 *4326GG and 412GG genotype. These patients experienced a significantly lower rate of response, with respect to patients harbouring the other genotypes.

Of note, the patients carrying both the *CYP1B1 *4326GG and the 4390AG or 4390GG genotype also experienced a significantly lower response rate with respect to all the patients harbouring the other genotypes. However, the patients carrying the *CYP1B1 *412GG and the 4390AG or the 4390GG genotype had similar response rate with respect to patients harbouring the other genotypes, suggesting that the 4326GG genotype might be the best pharmacogenetic marker of lower prevalence of response to docetaxel in CRPC patients.

Furthermore, the *CYP1B1 *4326GG genotype was associated with significantly shorter PFS and OS, whereas no correlations were observed between both the grouped *CYP1B1 *4326GG and 412GG and the grouped *CYP1B1 *4326GG and 4390AG or 4390GG genotypes with PFS.

Given the small number (N = 60) of patients enrolled in the study, in order to evaluate whether other prognostic factors could potentially explain their short survival, we performed corrections for multiple comparison and checked carefully several known baseline demographic, pathological and biochemical characteristics that predicted PSA decline and survival in previous studies in CRPC [27-29]. The occurrence of visceral metastasis and anaemia were associated with shorter OS, while age, ECOG-PS, and the docetaxel schedule were not associated with outcome. However, in the multivariate analysis, including all the significant variables from the univariate analysis, the *CYP1B1 *C4326C genotype remained an independent predictive parameter of death risk.

The underlying molecular mechanisms for the association between *CYP1B1 *4326GG genotype and the observed clinical outcome to docetaxel are not entirely clear. In view of previous studies demonstrating no correlation between docetaxel pharmacokinetics and *CYP1B1 *genotype [7], as well as *in vitro *studies indicating that docetaxel is not directly metabolized by CYP1B1 [30], this association could most likely be caused by indirect interactions. The CYP1B1-mediated 17β-estradiol metabolites, such as the catechol estrogens, quinonal and semiquinonal catechol estrogens, and methoxyestrogens can bind to tubulin at colchicine binding site [31,32], and can interfere with the microtubule stabilizing effect of docetaxel. This hypothesis is supported by a previous work by Sissung et al. [11], showing that the 4-OHE2-derived estrogen quinone completely abrogates the polymerization of tubuline. CYPY1B1-mediated estrogen metabolites that bind tubulin are more prevalent in the 4326GG genotype, and CRPC patients with this genotype had a significantly shorter OS. These results were further supported by a case report of a man with residual disease after radical prostatectomy, treated successfully with docetaxel. After only two cycles of therapy, a complete remission was obtained and then consolidated with additional cycles of docetaxel and radiotherapy. Prospective genetic analysis showed that this patient was heterozygous for the 4326CG (432LeuVal) polymorphism [33].

However in the study by Sissung et al. [11] no association was observed between 4326GG genotype and time-to-progression. In contrast, breast cancer patients carrying at least one C allele experienced a significantly shorter PFS following high-dose paclitaxel-based combination chemotherapy [34]. Similarly, in our study, significant correlations were observed between PFS and OS in patients grouped according to the 4326C>G polymorphism. However no reproducible significant associations between genotype and outcome were found for *CYP1B1 *polymorphism in a recent study in ovarian cancer patients treated with carboplatin and taxane regimens in the Scottish Randomised Trial in Ovarian Cancer phase III trial [23]. These controversial results suggest that pharmacogenetic associations may not always be reproducible when explored in small size series, without standardized unbiased methods, as well as in different settings for tumour type, stage and treatment. Further studies in larger homogeneous populations are warranted in order to understand the utility of candidate polymorphisms in the prediction of outcome in specific clinical settings.

Given the high variant allele frequencies of these polymorphisms, the statistical power to detect associations with these SNPs and response is greatly improved. Furthermore, we used very conservative statistical methods and correction for multiple comparisons to evaluate these relationships and reduce the chances of spurious findings. However, as most previous studies in this cancer setting, the present research is limited by the small sample and residual confounding could have resulted in our findings. Larger prospective studies are needed to validate these preliminary results, after adjustment for known prognostic factors. Furthermore, our data can only suggest a prognostic role for the *CYP1B1*-4326GG polymorphism, while its predictive role in docetaxel resistance should be evaluated in a prospective randomized study including a non-docetaxel containing arm.

## Conclusions

The data from the present investigational study provide the first evidence that the *CYP1B1*-4236GG polymorphism is linked to the clinical response to docetaxel, and may represent a potential new tool for treatment optimization. These results should prompt to perform multicenter prospective clinical trials, to validate *CYP1B1 *polymorphisms as possible biomarkers of docetaxel activity in CRPC patients.

## Competing interests

The authors declare that they have no competing interests.

## Authors' contributions

IP and EG participated in the design of the study, performed the experiments and the statistical analysis and drafted the manuscript. AC and FC participated in the analysis of the clinical and experimental data. CO participated in the acquisition and statistical analysis of clinical data. CC and SR were involved in the enrollement of the patients. RD, TSM, DKP, SR and WF participated in the conception, design, and coordination of the study. IP, EG, AC and TSM revised the manuscript critically. All authors have read and approved the final manuscript.

## Pre-publication history

The pre-publication history for this paper can be accessed here:

http://www.biomedcentral.com/1471-2407/10/511/prepub
